# Analysis of altmetrics and additional metrics of the 50 most-cited Behçet articles of all time: A Web of Science-based study

**DOI:** 10.22336/rjo.2022.59

**Published:** 2022

**Authors:** Cem Evereklioglu, Hidayet Sener, Fatih Horozoğlu

**Affiliations:** * Department of Ophthalmology, Erciyes University Medical Faculty, Kayseri, Turkey

**Keywords:** Behçet, altmetric attention score, impact factor, eigenfactor score, immediacy index

## Abstract

**Objective:** The number of citations is used for the scholarly impact of a published article, but it does not always correlate with higher-quality research. Behçet’s disease (BD) is a debilitating-blinding disorder with a significant volume of published articles in literature. Our aim was to investigate the references of the 50 most-cited Behçet articles and to evaluate the relationship between altmetric attention score (AAS) and additional metrics.

**Methods:** The Web of Science (WoS) core collection was used to search for the 50 most-cited Behçet articles. Additional metrics and AAS of the reviewed articles and the journals in which the articles were published, were evaluated.

**Results:** A total of 11.372 published articles on BD, between 1975 and 2022, were found. The citation range of 50 highly-cited articles was between 172 and 1322. The “top 50 list” articles were published between 1988 and 2018, and the average age of the article since publication was 18.86 ± 6.08 years. Rheumatology journals were the most published category with 21 articles. There was a weak-to-moderate correlation between AAS and additional metrics.

**Conclusions:** This is the first analysis regarding the AAS of the 50 most-cited articles on BD, which provides useful information about the social impact and characteristics in the academic community. AAS correlates weakly with citation-based quality indexes, and moderately with immediacy index, which evaluates speed. The publication year should be considered when comparing or evaluating the AAS of articles. AAS could be evaluated in a secondary plan for scientific impact analysis.

## Introduction

Since the first bibliographic publication of Garfield in JAMA (1987), various journals on numerous topics have reported the “50 most-cited articles” both in general and specific journals [**1**-**4**]. Bibliographic analyses are the evaluation of publications produced by individuals or institutions in a specific area, in a particular period, and in a precise region. Traditional metrics measure the impact of research in the scientific literature and are not important outside of the academic community [**5**]. However, it takes time to see their effects, and the rapid development of the internet and the electronic transformation of the academic publishing industry have resulted in the emergence of alternative metrics based on social networking activities [**6**]. Altmetrics is designed to track and measure the effect of research online and it collects information about articles from social media channels such as Twitter, Facebook, Google+, News, and Wikipedia. Each media source has a weighted score (**Table 1**, **Table 2**), and the altmetric attention score (AAS) calculated accordingly is presented in the altmetric donut with different colors [**7**].

**Table 1:** Weighted Count of the Altmetric Attention Score

News	8
Blog	5
Policy document (per source)	3
Patent	3
Wikipedia	3
Peer review (Publons, Pubpeer)	1
Weibo (not trackable since 2015, but historical data kept)	1
Google+ (not trackable since 2019, but historical data kept)	1
F1000	1
Syllabi (Open Syllabus)	1
LinkedIn (not trackable since 2014, but historical data kept)	0.5
Twitter (tweets and retweets)	0.25
Facebook (only a curated list of public Pages)	0.25
Reddit	0.25
Pinterest (not trackable since 2013, but historical data kept)	0.25
Q&A (Stack Exchan)	0.25
Youtube	0.25
Number of Mendeley readers	0
Number of Dimensions and Web of Science citations	0

**Table 2:** Metrics and characteristics of the Top 50 articles

	N	Times cited of WoS	Altmetric attention score	Publication year	NYsP	ACpY of WoS
All articles	50	236.5 (195.75-336.75)	3 (0-6)	2003 – 14 ± 6.08	18.86 ± 6.08	13.58 (10.07-19.38)
Study Type						
Systematic review and meta analyzes	2	230.5 (NA)	0 (NA)	2010 – ± 1.41	12 ± 1.41	19.2 (NA)
Reviews	14	219.5 (184-288.5)	3 (0-5.25)	2004 – 4 ± 4.75	17.5 ± 4.78	14.69 (11.61-17.56)
Original research	31	237 (196-377)	3 (0-6)	2001 – 4 ± 5.94	20.5 ± 5.94	13.05 (9.71-17.95)
Cohort study	10	240.5 (200.75-518.5)	3 (0-4)	2000 – 1 ± 6.95	21.9 ± 6.95	12.36 (9.35-24.19)
Observational study	9	236 (185.5-315.5)	0 (0-4.5)	1999 - ± 5.31	23 ± 5.31	10.24 (8.13-15.28)
Experimental study	5	229 (196-328)	6 (0-6)	2002 - ± 3.16	20 ± 3.16	13.47 (9.07-16.72)
Randomized controlled study	4	223 (199.25-307.5)	3 (3-7.5)	2001 – 5 ± 2.88	20.5 ± 2.88	17.79 (11.17-13.41)
Case control study	3	380 (NA)	12 (NA)	2011 - ± 1.73	11 ± 1.73	37.25 (NA)
Conference paper	2	380.5 (NA)	66.5 (NA)	2013 - ± 7.07	9 ± 7.07	51.82 (NA)
Letter to the editor	1	188	3	2004	18	10.44
Study Topic						
Full review	6	266 (192.5-581)	3 (0-34.25)	2002 – 6 ± 7.71	19.3 ± 2.5	12.77 (9.32-60.36)
Epidemiology	4	245 (190.25-423.5)	1.5 (0-5.25)	1998 - ± 4.96	24 ± 4.96	11.59 (8.52-14.86)
Pathogenesis	10	268 (229.5-416.5)	3.5 (2.25-7.75)	2004 - 4 ± 6.22	17.6 ± 6.22	17.4 (12.34-20.92)
Genetic	4	224.5 (182-324)	0.5 (0-7)	1999 - ± 8.67	23 ± 8.67	13 (6.61-15.91)
Diagnosis	2	344.5 (NA)	8.5 (NA)	2004 – 5 ± 4.94	17.5 ± 4.94	22.17 (NA)
Treatment	16	228 (189.75-367.75)	3 (0-5.75)	2003 – 3 ± 5.25	18.68 ± 5.25	13.63 (9.9-20.47)
Clinic presentation	8	215.5 (192.25-272)	1.5 (0-3.75)	2005 – 8 ± 4.96	16.1 ± 4.96	13.89 (9.48-19.52)
Median (25%-75% interquartile range), Mean ± Standard deviation, NA = not applicable, ACpY = average citation per year, NYsP = number of years since publication, WoS = Web of Science						

Behçet’s disease got its name from the Turkish dermatologist Hulusi Behçet, who first described the three-symptom-complex of oral aphthae, genital ulcer, and hypopyon uveitis in one of his patients in 1924 and published it in 1937 [**8**,**9**]. Behçet’s disease is widely seen among people living on the historical silk road, which is an old trade route, and is a chronic, recurrent, and multisystemic oro-facial mucocutaneous disease with or without hypopyon uveitis [**8**-**10**]. Gastrointestinal, central nervous system, or vascular involvements may also be encountered and the most important risk factor is HLA-B51 positivity [**11**]. Although Behçet’s disease is a rare condition in European countries, warnings have been issued about the possibility of an increase in the number of cases [**12**].

The importance of oro-facial mucocutaneous disorders with sight-threatening ocular complications is increasing and a significant number of articles are published in literature day by day. Therefore, the prompt diagnosis and appropriate referral to a related physician of patients with such a debilitating-blinding syndrome are vital for timely management. However, there is no article to model the contribution and importance level of various metrics to AAS about Behçet’s disease. This paper provides an overview of references analysis of the 50 most-cited articles on Behçet’s disease of all time (1975-2022) according to the Web of Science (WoS) database and presents significant knowledge on publishing researchers, journals, and various disciplines on Behçet’s disease.

## Materials and methods

WoS is a reliable scientific database and valuable information such as article citation statistics, authors, and country statistics can be accessed in this database. Therefore, we used WoS core collection database to search for the term “TS=(Behçet)”. As a result, fifty most-cited articles were included in the study (Date accessed: *February,15th 2022*). Time filter and other filters were not applied. Because the metrics of the articles according to the 50 most-cited papers was decreasing and similar studies used the first 50 most-cited articles in the analysis, we also selected the first 50 articles in the present research (**Table 3**, **Table 4**) [**2**-**4**]. All data obtained were recorded on the date of access. Irrelevant articles and papers whose relevant metrics could not be reached were excluded from the study. 

**Table 3:** Journals evaluated in the study

Journal Name	Number of Articles	Impact Factor	H Index	Q Category
JOURNAL OF RHEUMATOLOGY	5	4,666	178	Q2
ARTHRITIS AND RHEUMATISM	4	10,995	314	Q1
NATURE GENETICS	3	38,33	573	Q1
SEMINARS IN ARTHRITIS AND RHEUMATISM	3	5,532	114	Q1
ANNALS OF INTERNAL MEDICINE	2	25,391	390	Q1
ANNALS OF THE RHEUMATIC DISEASES	2	19,103	240	Q1
BRAIN	2	13,501	336	Q1
RHEUMATOLOGY	2	7,58	173	Q1
JOURNAL OF NEUROLOGY	2	4,849	136	Q1
INTERNATIONAL JOURNAL OF DERMATOLOGY	2	2,736	93	Q3
NEW ENGLAND JOURNAL OF MEDICINE	1	91,253	1.030	Q1
LANCET	1	79,323	762	Q1
LANCET NEUROLOGY	1	44,182	291	Q1
NATURE CLINICAL PRACTICE RHEUMATOLOGY	1	20,543	137	Q1
PROCEEDINGS OF THE NATIONAL ACADEMY OF SCIENCES OF THE UNITED STATES OF AMERICA	1	11,205	771	Q1
ARCHIVES OF DERMATOLOGY	1	10,995	166	Q1
AUTOIMMUNITY REVIEWS	1	9,754	122	Q1
JOURNAL OF AUTOIMMUNITY	1	7,094	113	Q1
JOURNAL OF THE EUROPEAN ACADEMY OF DERMATOLOGY AND VENEREOLOGY	1	6,166	107	Q1
SURVEY OF OPHTHALMOLOGY	1	6,048	132	Q1
AMERICAN JOURNAL OF OPHTHALMOLOGY	1	5,258	186	Q1
INVESTIGATIVE OPHTHALMOLOGY & VISUAL SCIENCE	1	4,799	218	Q1
ARTHRITIS CARE & RESEARCH	1	4,794	163	Q2
MEDIATORS OF INFLAMMATION	1	4,711	97	Q2
BRITISH JOURNAL OF OPHTHALMOLOGY	1	4,638	153	Q1
TISSUE ANTIGENS	1	4,513	99	Q3
CLINICAL AND EXPERIMENTAL RHEUMATOLOGY	1	4,473	95	Q2
CLINICAL AND EXPERIMENTAL MEDICINE	1	3,984	45	Q3
AMERICAN JOURNAL OF NEURORADIOLOGY	1	3,825	177	Q2
SCANDINAVIAN JOURNAL OF RHEUMATOLOGY	1	3,641	77	Q3
INFECTION AND IMMUNITY	1	3,441	220	Q3
CLINICAL RHEUMATOLOGY	1	2,980	82	Q3
MEDICINE	1	1,889	148	Q3

**Table 4:** Journal metrics and changes by Q category of WoS and metrics by journal category

Journal Category	N	Times cited of WoS	Altmetric attention score	Journal citation indicator	Impact factor	H index	Normalized eigenfactor score	Immediency index	Article influence score
All Journals	50	236.5 (195.75-336.75)	3 (0-6)	1.49 (1.01-3.02)	5.79 (4.66-13.5)	177.5 (114-314)	4.50 (3.05-12.88)	2.97 (1.58-6.27)	1.954 (1.33-4.81)
Q1 category	33	236 (195.5-365)	3 (0-6.5)	2.70 (1.49-5.05)	10.99 (5.79-22.96)	240 (136-363)	8.96 (4.26-15.32)	4.48 (2.41-13.67)	3.37 (1.95-9.08)
Q2 category	9	251 (183-288)	0 (0-1.5)	1.08 (0.87-1.08)	4.66 (4.56-4.68)	177.5 (114-314)	3.54 (3.54-4.08)	2.97 (1.63-2.97)	1.34 (1.21-1.34)
Q3 category	8	235 (180.75-288.25)	3 (0.75-3)	0.73 (0.57-0.86)	3.21 (2.73-3.89)	93 (78.25-135.75)	1.55 (0.48-3.63)	0.67 (0.57-0.90)	1.22 (0.74-1.58)
Rheumatology	21	229 (194.5-277.5)	0 (0-4)	1.08 (1.01-2.7)	5.53 (4.66-10.99)	178 (114-240)	3.54 (2.72-8.96)	2.97 (1.58-4.48)	1.95 (1.34-3.37)
Neurology	6	218.5 (189.75-330.25)	3.5 (0-6.25)	2.25 (1.15-4.88)	9.17 (4.59-21.17)	234 (136-336)	8.74 (4.53-12.88)	3.17 (2.02-7.87)	3.17 (1.37-7.21)
General Medicine	6	354 (185.5-715.25)	3 (2.25-6.25)	6.28 (0.83-21.57)	25.39 (3.46-82.3)	390 (122.25-829)	19.47 (12.98-103.05)	40.55 (0.99-186.28)	11.42 (0.63-31.13)
Immunology	5	196 (182-252.5)	0 (0-3.5)	0.9 (0.57-1.3)	4.71 (3.97-8.42)	113 (98-171)	3.1 (1.46-4.06)	5.75 (0.71-13.67)	1.12 (0.93-2.02)
Dermatology	4	235 (202.5-504.5)	3 (3-10.5)	1.21 (0.72-2.45)	4.45 (2.73-9.78)	100 (93-151.25)	2.81 (1.55-4.38)	3.14 (1.58-6.47)	0.99 (0.57-2.82)
Ophthalmology	4	261.5 (205.25-364.25)	6 (3.75-6)	1.65 (1.20-1.96)	5.02 (4.67-5.85)	169.5 (137.25-210)	4.97 (1.87-9.24)	2 (0.94-2.73)	1.47 (1.32-1.76)
Genetics & Hereditery	3	380 (NA)	12 (NA)	8.78(NA)	38.33(NA)	573 (NA)	39.12 (NA)	6.27 (NA)	19.01 (NA)
Multidisciplinary Sciences	1	334	3	2.04	11.2	771	168.93	3.16	4.79
Median (25%-75% interquartile range), NA = not applicable									

Titles, author information, publication year, number of years since publication (NYsP), number of citations, AAS, and average citations per year (ACpY) were recorded. The journals in which the articles were published, IF, 5-year IF, Q category, JCI, ES, AIS, II, and h-index were recorded. The AASs of the articles were recorded from the bookmark “Altmetric it!”. The h-index of journals was recorded from scientific journal rankings. All other metrics were recorded from the WoS and journal citation report 2020. 

Statistical analyzes were performed using with web-based software TURCOSA (www.turcosa.com.tr). Shapiro-Wilk test was used for normality. According to the distribution, mean ± standard deviation and median (first - 25% and third - 75% quartiles), were used. Spearman’s rho correlation analysis was performed. Significance levels were set at p < 0.05. Multivariate adaptive regression splines (MARS) was performed in Salford Predictive Modeller version 8 software to model AAS according to journal (5-year IF, normalized ES, AIS, II, h-index) and article (times cited of WoS, NYsP) metrics.

## Results

*Characteristics and Metrics of the Top 50 articles*

**Fig. 1:**
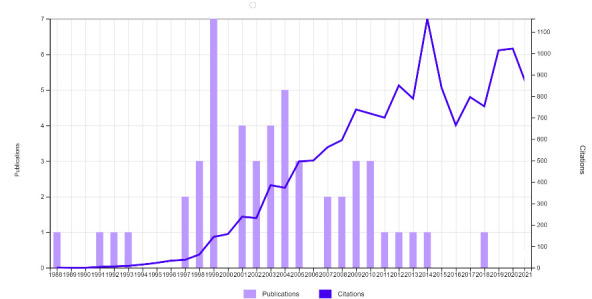
Cumulative display of citations by year and publication years of the articles included in the study

**Fig. 2:**
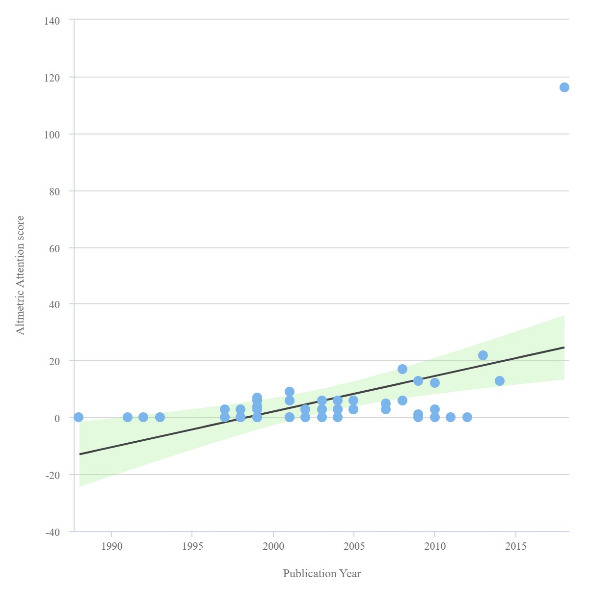
Linear regression graph showing the relationship between publication year and altmetric attention score of the articles included in the study

A total of 11.372 published articles were found on Behçet’s disease between 1975 and 2022. The citation range of the 50 highly-cited articles was between 172 and 1322. The top 50 listed articles were published between 1988 and 2018 and the average age of the article since the publication was 18.86 ± 6.08 years. The year of publication with the number of citations trend is presented in **Fig. 1**. The scatter plot of AAS by years is presented in **Fig. 2**. The most-cited article was “Current concepts - Behçet disease” written by Sakane T et al., which was published in “New England Journal of Medicine” in 1999. The AAS was between 0 and 116. The article with the highest AAS was “2018 update of the EULAR recommendations for the management of Behçet syndrome” written by Hatemi G et al., published in “Annals of the Rheumatic Diseases” in 2018. Yazici H was the author with the most published articles (11 publications). Sfikakis PP was the first author with 3 articles. The affiliation with the highest number of authors in the top 50 was Istanbul University (20 authors). Metrics of the included articles are presented in **Table 2**. 

*Characteristics of journals with the top 50 articles published and correlations between altmetric attention score and journal and article metrics*

The articles included in the study were published in 33 journals (**Table 3**). 

**Table 5:** Articles included in study

	Title	First Author	Years	Times cited
1	Current Concepts – Behcet’s Disease	Sakane T	1999	1322
2	The International Criteria For Behcet’s Disease (Icbd): A Collaborative Study Of 27 Countries On The Sensitivity And Specificity Of The New Criteria	Davatchi F	2014	590
3	Clinical Patterns Of Neurological Involvement İn Behcet’s Disease: Evaluation Of 200 Patients	Akman-Demir G	1999	535
4	The Long-Term Mortality And Morbidity Of Behcet Syndrome - A 2-Decade Outcome Survey Of 387 Patients Followed At A Dedicated Center	Kural-Seyahi E	2003	513
5	Eular Recommendations For The Management Of Behcet Disease	Hatemi G	2008	485
6	Vascular Involvement In Behcets-Dısease	Koc Y	1992	467
7	Genome-Wide Association Study Identifies Variants In The Mhc Class I, Il10, And Il23r-Il12rb2 Regions Associated With Behcet’s Disease	Remmers Ef	2010	447
8	Uveitis In Behcet Disease: An Analysis Of 880 Patients.	Tugal-Tutkun I	2004	390
9	Genome-Wide Association Studies Identify Il23r-Il12rb2 And Il10 As Behcet’s Disease Susceptibility Loci	Mizuki N	2010	380
10	Effect Of Infliximab On Sight-Threatening Panuveitis In Behcet’s Disease	Sfikakis Pp	2001	377
11	Behcet’s Disease	Kaklamani Vg	1998	353
12	Genome-Wide Association Analysis Identifies New Susceptibility Loci For Behcet’s Disease And Epistasis Between Hla-B*51 And Erap1	Kirino Y	2013	345
13	Triplet Repeat Polymorphism In The Transmembrane Region Of The Mıca Gene: A Strong Association Of Six Gct Repetitions With Behcet Disease	Mizuki N	1997	334
14	Thalidomide In The Treatment Of The Mucocutaneous Lesions Of The Behcet Syndrome - A Randomized, Double-Blind, Placebo-Controlled Trial	Hamuryudan V	1998	331
15	Overproductıon Of Monocyte-Derıved Tumor-Necrosıs-Factor-Alpha, Interleukın-(Il)-6, Il-8 And Increased Neutrophıl Superoxıde Generatıon In Behcets-Dısease - A Comparatıve-Study Wıth Famılıal Medıterranean Fever And Healthy-Subjects	Mege Jl	1993	297
16	Behcet’s Disease, The Silk Road And Hla-B51: Historical And Geographical Perspectives	Verity Dh	1999	293
17	Current Concepts In The Etiology And Treatment Of Behcet Disease	Evereklioglu C	2005	287
18	Efficacy, Safety, And Pharmacokinetics Of Multiple Administration Of Infliximab In Behcet’s Disease With Refractory Uveoretinitis	Ohno S	2004	279
19	2018 Update Of The Eular Recommendations For The Management Of Behcet’s Syndrome	Hatemi G	2018	276
20	Cytokine Profile In Behcet’s Disease Patients - Relationship With Disease Activity	Hamzaoui K	2002	274
21	Neuro-Behcet’s Disease: Epidemiology, Clinical Characteristics, And Management	Al-Araji A	2009	262
22	Behcet’s Disease: An Update On The Pathogenesis	Gul A	2001	256
23	Hla-B51/B5 And The Risk Of Behcet’s Disease: A Systematic Review And Meta-Analysis Of Case-Control Genetic Association Studies	De Menthon M	2009	251
24	Prevalence Of Behcet’s Disease In Istanbul, Turkey	Azizlerli G	2003	248
25	A Double-Blind Trial Of Colchicine In Behcet’s Syndrome	Yurdakul S	2001	237
26	Upregulated Il-23 And Il-17 In Behcet Patients With Active Uveitis	Chi W	2008	236
27	Neurological Complications In Behcet’s Syndrome	Kidd D	1999	233
28	Efficacy Of İnfliximab İn The Treatment Of Uveitis That İs Resistant To Treatment With The Combination Of Azathioprine, Cyclosporine, And Corticosteroids In Behcet’s Disease - An Open-Label Trial	Tugal-Tutkun I	2005	229
29	Anti-Tnf Therapy In The Management Of Behcet’s Disease - Review And Basis For Recommendations	Sfikakis Pp	2007	227
30	Evaluation Of Clinical Findings According To Sex In 2313 Turkish Patients With Behcet’s Disease	Tursen U	2003	222
31	Behcet’s Disease - A Contemporary Review	Mendes D	2009	212
32	Anti-Tnf Agents For Behcet’s Disease: Analysis Of Published Data On 369 Patients	Arida A	2011	210
33	Short-Term Trial Of Etanercept İn Behcet’s Disease: A Double Blind, Placebo Controlled Study	Melikoglu M	2005	209
34	Behcet’s Disease: Diagnostic And Prognostic Aspects Of Neurological Involvement	Siva A	2001	204
35	Behcet’s Syndrome: Disease Manifestations, Management, And Advances İn Treatment	Yazici H	2007	200
36	Azathioprine In Behcet’s Syndrome - Effects On Long-Term Prognosis	Hamuryudan V	1997	197
37	New Insights Into The Pathogenesis Of Behcet’s Disease	De Chambrun Mp	2012	196
38	Interferon Alfa-2a In The Treatment Of Behcet Disease - A Randomized Placebo-Controlled And Double-Blind Study	Alpsoy E	2002	196
39	Human Recombinant Interferon Alfa-2a For The Treatment Of Behcet’s Disease With Sight Threatening Posterior Or Panuveitis	Kotter I	2003	195
40	Th1 Polarization Of The Immune Response In Behcet’s Disease - A Putative Pathogenetic Role Of İnterleukin-12	Frassanito Ma	1999	192
41	Cns İnvolvement In Neuro-Behcet Syndrome: An Mr Study	Kocer N	1999	191
42	Assocıatıon Between The 65-Kılodalton Heat-Shock Proteın, Streptococcus-Sanguıs, And The Correspondıng Antıbodıes In Behcets-Syndrome	Lehner T	1991	189
43	Infliximab For Recurrent, Sight-Threatening Ocular İnflammation İn Adamantiades-Behcet Disease	Sfikakis Pp	2004	188
44	Behcet’s Disease And The Nervous System	Serdaroglu P	1998	186
45	Behcet’s Disease: Evaluation Of A New Instrument To Measure Clinical Activity	Bhakta Bb	1999	182
46	Behcet’s Disease	Kurokawa Ms	2004	178
47	The Use Of İnterferon Alpha In Behcet Disease: Review Of The Literature	Kotter I	2004	177
48	Behcet’s Disease: From East To West	Davatchi F	2010	176
49	Serum Levels Of Tnf-Alpha, Sıl-2r, Il-6, And Il-8 Are Increased And Associated With Elevated Lipid Peroxidation İn Patients With Behcet’s Disease	Evereklioglu C	2002	175
50	The Prevalence Of Behcet Syndrome In A Rural Area In Northern Turkey	Yurdakul S	1988	172

The journals were all SCI-Expanded. The most published journal category was rheumatology, with 21 articles. The median citation and AAS were 229 (194.5-277.5) and 0 (0-4), respectively. “Journal of Rheumatology” was the journal with the most articles in the list with 5 articles and the median citations of WoS and AAS were 256 (216.5-332.5) and 3 (1.5-3), respectively. Springer Nature and Elsevier were the publishers with the most articles, with 8 articles each. Journal metrics are presented in **Table 4**. Citation and AAS evaluation by journal clinical category are presented in **Table 4**.

**Fig. 3:**
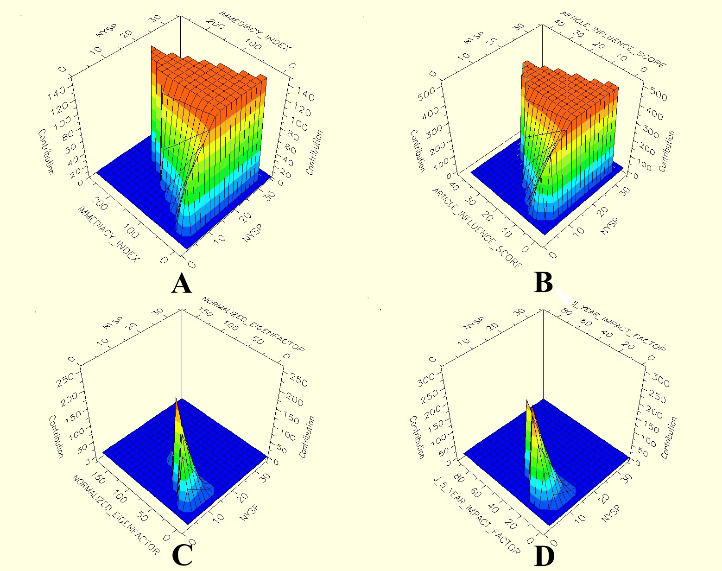
Three-dimensional representation of the contribution of the interaction of NYsP between **(A)** immediacy index, **(B)** article influence score, **(C)** normalized eigenfactor score, **(D)** 5-year impact factor to the MARS model

There was a weak-to-moderate correlation between AAS and additional metrics. The correlation between AAS and journal metrics and article metrics is presented in **Table 6**. The importance score of explanatory variables according to the MARS model is as follows: NYsP (100%), article influence score (50.5%), normalized eigenfactor score (46.76%), the 5-year impact factor (37.67%), and immediacy index (5.41%). Predictive variables were listed according to their importance scores calculated on the 100% scale. The most important variable always received a 100% score. Adjusted R2 with MARS model was 0.96. The contribution of the interaction of predictive variables to AAS is presented in **Fig. 3**. This figure represents the contribution level, which is the model of binary interactions.

**Table 6:** Correlation between AAS and article and journal metrics

Metrics	Spearman rho	p value
AAS – Times cited of WoS	0.359	0.010*
AAS – Times cited of All databases	0.398	0.004*
AAS – Publication year	0.338	0.005*
AAS – NYsP	0.338	0.005*
AAS – ACpY of WoS	0.463	0.001*
AAS – ACpY of All databases	0.469	0.001*
AAS – Impact Factor	0.393	0.005*
AAS – 5-year impact Factor	0.388	0.005*
AAS – H Index	0.303	0.033*
AAS – Journal Citation Indicator	0.476	<0.001*
AAS – Eigenfactor score	0.368	0.009*
AAS – Normalized Eigenfactor score	0.368	0.009*
AAS – Article Influence score	0.355	0.011*
AAS – Immediacy Index	0.411	0.003*
AAS – % citable of open access	0.183	0.202
AAS = Altmetric attention score, ACpY = Average citation per year, NYsP = Number of years since publication, WoS = Web of Science, significance level is p <0.05		

## Discussion

One of the most important features of altmetrics is its speed and the AAS can reflect the zeitgeist of the moment [**13**]. Even if an article makes a significant contribution to its field, it may take a long time to be cited (**Fig. 2**) [**14**]. When we filtered the articles according to years, the article published in 1999 was the most cited, but the article published in 2018 had the highest AAS. This can be explained as a reflection of the increase in the use of social media on the AAS over the years [**15**]. The other possible explanation may be directly related to the rise in the use of academic social networking platforms by researchers, along with the launch of a company providing altmetric data in 2011 [**16**]. In our article pool, the mean age of the articles was 18.86 ± 6.08 years, the mean year of publication was 2003, 14 ± 6.08, and the rate of mentioning old articles on social media was low [**17**]. Therefore, the median of AAS of our top 50 list may be low. 

Weak to moderate correlations were found between AAS and citation and citation-based metrics. However, AAS may exhibit exponential growth concurrent with the increase in the use of network platforms, especially in recent years. For this reason, the MARS model, which can establish both a linear and nonlinear model, was used while creating the regression model to evaluate the relationship between AAS and additional metrics. The fact that NYsP is the first most important predictor in the regression model can be explained by the increased circulation of new articles in the online world due to the increase in the usage of such networks. The fact that citation-based metrics were following NYsP indicates the importance of publishing an article in top journals. However, citation-based metrics interacted with the NYsP and contributed to the model, emphasizing the importance of the NYsP.

Journals publishing in the category of genetics have had higher AAS in recent years [**14**]. This situation did not change in the case of Behçet’s disease, and journals in the genetic and ophthalmology category have the highest AAS. Journals in the genetic and general medicine category had the highest additional metric scores in our study. What should be understood when interpreting AAS is that the AAS does not inform about the quality of the article, it just follows attention. Positive or negative feedback will be reflected in the AAS as plus points. Additional metrics are available based on citations of articles. There was a weak correlation between the eigenfactor score and article influence score, which are indexes that evaluate quality, and AAS. There was a moderate correlation between AAS and immediacy index in our study. These additional metrics are available in the AAS explanatory model, but these metrics represent the quality of the journal and in our series, the most important variable explaining AAS is the NYsP.

Turkey has the highest prevalence of Behçet’s disease (370-420 cases/ 100,000) whereas in Japan, Korea, China, Iran, and Saudi Arabia the prevalence is 13.5-22 cases/ 100,000 [**18**-**20**]. In the present study, we evaluated the research outputs and the journals related to Behçet’s disease using bibliometric methods. 

There are a few studies that perform bibliographic analysis on Behçet’s disease. Masri et al. [**21**] assessed the numerical contribution of the Arab world to research on Behçet’s disease using the PubMed platform between 2005 and 2019, whereas Şenel et al. [**22**] investigated productivity performances of 78 countries between 1980 and 2014, and finally Kocyiğit et al. [**23**] evaluated Behçet articles only between 2010 and 2019. However, either an altmetric analysis was not performed, or only a country-based altmetric analysis was investigated in all these three articles. Similarly, they used a limited period of time in their studies. In the present paper, we aimed to evaluate the first 50 articles of all time, between 1975 and 2022, which drew the attention of the scientific community and received the most citations, at the same time evaluating the relationship between AAS and various additional metrics. The author with the most published articles and the affiliation placing in the “top 50 list” in our study was from Turkey. The first author with the most published articles was from Greece, and the most-cited article was from Japan. It is not surprising that the articles were produced in these countries with a high prevalence of Behçet’s disease, indicating an increasing number of publications in the investigation productivity from the abovementioned silk-road countries. The findings of Kocyigit et al. [**23**] support our hypothesis that the countries with the highest number of articles on Behçet’s disease are located on the silk road and that Turkey is the country that produces the most articles.

 Behçet’s disease is not only a disease characterized by oro-facial mucocutaneous lesions and hypopyon uveitis, but also many organs and systems are involved. Therefore, multidisciplinary evaluations are essential among many physicians [**24**], and a collaborative approach enables the early diagnosis of various organ involvements and therefore allows a prompt treatment outcome [**25**]. Although there are articles from journals in different categories in our review, rheumatology journals dominate the “top 50 list” with 21 articles, which was similar to the study of Kocyigit et al. [**23**]. This may be because Behçet patients are followed up in rheumatology clinics mostly and referred to other clinics according to their symptoms. In other words, it may be because the chief is a rheumatologist in the multidisciplinary approach. Another reason may be that the authors prefer rheumatology journals. 

The treatment of Behçet’s disease is complex because multiple drugs are required to target various organ systems. There may be differences in the medical management of mucocutaneous, joint, eye, vascular, neurological, and gastrointestinal involvement of Behçet disease [**26**,**27**]. In the present article, there were 16 articles on the “treatment” topic and 10 articles on the “pathogenesis” topic. When these topics were reviewed, it was understood that there was an increasing importance of inflammatory cytokines in Behçet’s disease and the use of biological agents in the treatment. However, the “diagnosis” topic was the highest with an AAS of 8.5. This may be related to the low number of articles on this topic and the articles were more up-to-date. On the other hand, when the articles were evaluated according to the study type, our results showed that the AAS of the systematic review and meta-analysis, which were considered to be the studies with the highest level of evidence and valuable in the scientific community, did not receive the expected attention in the social media.

The strength of this study was that we did not limit the category of the journal and the time range. The limitation of this study was that we only used the WoS database. The fact that Behçet’s disease is common in some regions, but is a rare disease in other areas, might have affected the AAS. Studies on popular topics (e.g. coronavirus, zikavirus) might have high altmetric scores and future studies on the effect of AAS popularity might be performed.

## Conclusion

In conclusion, this is the first analysis of the AAS of the 50 most-cited articles on Behçet’s disease. It provides useful information about the social impact and characteristics of the 50 most-cited studies on Behçet’s disease in the academic community. AAS correlated weakly with the citation-based quality indexes, and moderately with immediacy index, which evaluates speed. The publication year should be considered when comparing or evaluating the AAS of articles. AAS could be evaluated in a secondary plan for scientific impact analysis.

**Conflict of Interest statement**

The authors state no conflict of interest.

**Acknowledgements**

None.

**Sources of Funding**

None.

**Disclosures**

None.
